# CD45RB Glycosylation and Ig Isotype Define Maturation of Functionally Distinct B Cell Subsets in Human Peripheral Blood

**DOI:** 10.3389/fimmu.2022.891316

**Published:** 2022-04-28

**Authors:** Jana Koers, Sabrina Pollastro, Simon Tol, Ingrid Pico-Knijnenburg, Ninotska I. L. Derksen, Pauline A. van Schouwenburg, Mirjam van der Burg, S. Marieke van Ham, Theo Rispens

**Affiliations:** ^1^ Landsteiner Laboratory, Sanquin Research, Department of Immunopathology, Amsterdam UMC, University of Amsterdam, Amsterdam, Netherlands; ^2^ Landsteiner Laboratory, Sanquin Research, Department of Research Facilities, Amsterdam University Medical Center (UMC), University of Amsterdam, Amsterdam, Netherlands; ^3^ Laboratory for Pediatric Immunology, Department of Pediatrics, Leiden University Medical Center, Leiden, Netherlands; ^4^ Swammerdam Institute for Life Sciences, University of Amsterdam, Amsterdam, Netherlands

**Keywords:** CD45RB glycosylation, CD27, B cell subset heterogeneity, naive B cells, memory B cells

## Abstract

Glycosylation of CD45RB (RB+) has recently been identified to mark antigen-experienced B cells, independent of their CD27 expression. By using a novel combination of markers including CD45RB glycosylation, CD27 and IgM/IgD isotype expression we segregated human peripheral blood B cell subsets and investigated their IGHV repertoire and *in vitro* functionality. We observed distinct maturation stages for CD27-RB+ cells, defined by differential expression of non-switched Ig isotypes. CD27-RB+ cells, which only express IgM, were more matured in terms of Ig gene mutation levels and function as compared to CD27-RB+ cells that express both IgM and IgD or cells that were CD27-RB-. Moreover, CD27-RB+IgM+ cells already showed remarkable rigidity in IgM isotype commitment, different from CD27-RB+IgMD+ and CD27-RB- cells that still demonstrated great plasticity in B cell fate decision. Thus, glycosylation of CD45RB is indicative for antigen-primed B cells, which are, dependent on the Ig isotype, functionally distinct.

## Introduction

B cell development and the formation of a highly diverse pool of long-lived antibody-secreting cells and memory B cells is crucial for the humoral immune response and lasting immunological memory. During the course of an immune response, the immunoglobulin (Ig) gene is changed by the process of somatic hypermutation (SHM), class switch recombination (CSR), and selection in response to antigen stimulation. Hypermutated and class-switched Ig genes are a characteristic of memory B cells along with the loss of IgD expression. Gain of CD27 expression, often combined with absent IgD expression, is commonly used to discriminate human memory B cells from naive B cells. However, a substantial proportion of CD27- B cells in healthy adults displays mutated and isotype-switched Ig genes like memory B cells (1–3), which disputes the feasibility of using CD27 as a truly discriminative memory marker. Mutated CD27- cells are present at birth (4) and account for 5% of the peripheral blood B cell population in healthy adults (1). The function of these putative CD27- memory cells and their relationship to CD27+ cells is not well understood but increased frequencies have been observed during aging (5), in autoimmunity (6) and several viral infections (7, 8). The lack of markers to distinguish naive B cells from memory B cells resulted in novel strategies for B cell classification using phenotypical differences in addition to CD27 and Ig isotype (9). Several studies identified changes in site-specific O-linked glycosylation of CD45RB during human B cell differentiation in various secondary lymphoid organs and blood (10–14). This glycan-dependent CD45RB epitope can be detected using the mAb MEM-55 and within human peripheral blood, glycosylation of CD45RB (RB+) was absent on naive B cells, but highly expressed on CD27+ cells and on a small fraction of switched and non-switched CD27- B cells (10). Next-generation sequencing of the Ig heavy chain variable (IGHV) genes revealed that these CD27-RB+ cells displayed intermediate levels of somatic mutations, higher than CD27-RB- but lower than CD27+RB+ cells (1, 14), suggesting that these B cells are antigen-primed and no longer naive. On the other hand, these CD27-RB+ cells, with high IgM levels, were found with increased frequencies in young children and early after hematopoietic stem cell transplantation (HSCT) (11), suggesting an immature rather than memory stage. Thus, CD45RB glycosylation, in combination with CD27 expression and Ig isotype, may serve as an additional marker to improve classification of human naive and memory B cells. Here, we investigated human peripheral blood B cell subsets segregated by CD45RB glycosylation, CD27 and IgM/IgD isotype expression and studied their IGHV repertoire and *in vitro* functionality.

## Materials and Methods

### Human B Cell Isolation

Buffy coats were obtained from anonymized adult healthy donors with written informed consent in accordance to the guidelines established by the Sanquin Medical Ethical Committee and in line with the Declaration of Helsinki. Human peripheral blood mononucleated cells (PBMCs) were isolated from buffy coats using Ficoll gradient centrifugation (lymphoprep; Axis-Shield PoC AS) and CD19^+^ cells were isolated by positive selection using magnetic Dynabeads (Invitrogen).

### FACS Sorting

Cryopreserved CD19+ cells were surface stained in PBA (PBS supplemented with 0.1% bovine serum albumin) for 30 min at 4°C and washed in PBA. A LIVE/DEAD cell staining (ThermoFisher scientific) was used to label and exclude non-viable cells. B cells were gated as singlet, viable, CD19+ cells and were segregated further into six subsets using surface marker expression of: CD27, CD38, CD45RB glycosylation, IgD, IgM, IgG, and IgA. Subsets were FACS-sorted (FACS Aria III, BD Biosciences) into RTL buffer (Sigma-Aldrich) for next-generation sequencing or in PBA for *in vitro* stimulation assays.

### Antibodies

CD19^+^ cells were surface stained using the following antibodies: anti-CD19 (clone SJ25C1, 562947), anti-CD38 (clone HB7, 646851), anti-IgD (clone IA6, 2561315), anti-IgM (clone G20-127, 562977) from BD Biosciences. Anti-IgG (MH16-1, M1268) from Sanquin Reagents. Anti-IgA (polyclonal, 2050-09) from SouthernBiotech. Anti-CD27 (clone O323, 25-0279-42) from ThermoFisher. Anti-CD45RB (clone MEM-55, 310205) from Biolegend.

### Immune Repertoire Sequencing

5000 B cells were sorted per subset per donor (n = 3), lysed and gDNA was extracted according to the manufacturer’s instructions (Qiagen). IgH rearrangements were amplified in a multiplex PCR using forward primers V_H_1-6 FR1 (BIOMED-2) and J_H_ consensus reverse primers with a Molecular Identifier (MID)-tag. A total of 40 PCR cycles were performed and a heat stable polymerase with proof-reading activity was used (AmpliTaq Gold DNA polymerase, Thermo Fisher). Equal quantities of PCR products were pooled and purified by gel extraction (MinElute gel extraction kit, Qiagen) and Agencourt AMPure XP beads (Backman Coulter) and quantified by fluorescence using the Qubit 2.0 Fluorometer (Invitrogen). Sequencing was performed using a Illumina Miseq run in standard mode using a v3 kit.

### Repertoire and Mutation Analysis

Reads retrieved from the sequencing platform were processed using pRESTO (15). Briefly, paired-ends reads were assembled using the AssemblePairs.py function and assembled reads with a phred score < 25 were removed. V_H_, J_H_ primers and MID-tags were annotated and masked using the MaskPrimers.py function. Obtained datasets were then submitted to IMGT/HighV-QUEST (16, 17) for alignment. Aligned sequences were further processed using Change-O (18). Output of the IMGT/HighV-QUEST was parsed using the MakeDb.py function (18) and only functional rearrangements were selected. Analysis of somatic hypermutation was performed using the SHazaM R package (version 0.2.1) after germline sequence reconstruction using the CreateGermlines.py function from Change-O. All data here presented are performed on a final dataset of unique nucleotidic sequences (spacing from the IGHV to the IGHJ gene, primers excluded). Due to the lack of unique molecular identifiers (UMIs) in the amplification protocol, it was not possible to properly correct for technical artifacts such as PCR and sequencing errors. Therefore, we removed reads that occurred only once (singletons) with the assumption that technical artifacts occur randomly and independently of each sample’s origin and are therefore equally distributed in all analyzed samples. Information on the total and unique rearrangements per sample, before and after singletons exclusion, and IGHV and IGHJ gene representation is reported in **Supplementary Table 1**. Raw sequencing data have been deposited at NCBI Sequence Read Archive (BioProject: PRJNA816414) and processed repertoires are available upon request to the corresponding author.

### *In Vitro* Stimulation Assays

*T cell dependent (TD) stimulation assays*. 96- well flat-bottom plates were seeded with 0.01x10^6^ irradiated 3T3 fibroblast cells expressing human CD40L per well in B cell medium (RPMI medium supplemented with FCS (5%, Bodinco), penicillin (100 U/mL, Invitrogen), streptomycin (100 μg/mL, Invitrogen), β-mercaptoethanol (50 μM, Sigma-Aldrich), L-glutamine (2mM, Invitrogen), and human apo-transferrin [20 μg/mL, Sigma-Aldrich] depleted for IgG using protein A sepharose (GE Healthcare)) and adhered overnight. The generation of CD40L expressing fibroblasts is described elsewhere (19). The next day, B cell medium supplemented with recombinant human IL-21 (50ng/ml, Preprotech) or IL-21 and IL-4 (50ng/ml and 25ng/ml, Preprotech) was added to the seeded 3T3-CD40L cells. B cell subsets (**Figure 2A**) were directly sorted into the plates in a density of 250 cells/well. After ten days of culture, survival, isotype switching, differentiation and Ig secretion was assessed using flow cytometry and ELISA.

*T cell independent (TI) stimulation assays*. 96- well flat-bottom plates were seeded with 10.000 B cells from each subset per well supplemented with either 0.1μM CpG ODN (*In vivo*gen) or 1μM R848 (*In vivo*gen), in the presence of 1μg/ml anti-IgM F(ab’)_2_ (JacksonImmunoResearch). After seven days of culture B cell survival, isotype switching, differentiation and Ig secretion were assessed using flow cytometry and ELISA.

### IgM, IgG, and IgA ELISA of Culture Supernatants

IgM, IgG, and IgA expression levels were measured as described previously (19). In brief, 96-well maxisorb plates were coated with monoclonal anti-IgM, anti-IgG, or anti-IgA (2μg/ml MH15-1, 2μg/ml MH16-1, or 1μg/ml MH14-01-M08, Sanquin reagents) and culture supernatants were incubated for 1h. Secreted IgM, IgG, or IgA was detected using HRP-conjugated mouse anti-human IgM, IgG, or IgA (1μg/ml MH15-1, 1μg/ml MH16-1, or 1μg/ml MH14-1, Sanquin reagents) in HPE (Sanquin reagents). The ELISA was developed using TMB substrate, stopped by addition of 0.2M H_2_SO_4_ and absorbance was measured at 450 and 540 nm. OD values were normalized to values of a reference serum pool that was included in each plate. To properly determine IgM production, *in vitro* stimulation assays described above, were also performed in the absence of anti-IgM F(ab’)_2_ as this compound interferes with the IgM ELISA resulting in an incomplete picture of the IgM production. Of note, the presence of anti-IgM F(ab’)_2_ may alter IgM production during culture.

### Statistical Analysis

Differences between groups were analyzed using a Friedman analysis of variance for non-parametric repeated measures followed by a Dunn’s multiple comparison test. A *p* value <0.05 was considered significant. The statistical analysis was carried out using GraphPad Prism 9.1.1.

## Results

### B Cell Subsets Separated Based on CD45RB Glycosylation and CD27 Expression Feature Differential Ig Isotype Usage

**Figure 1:**
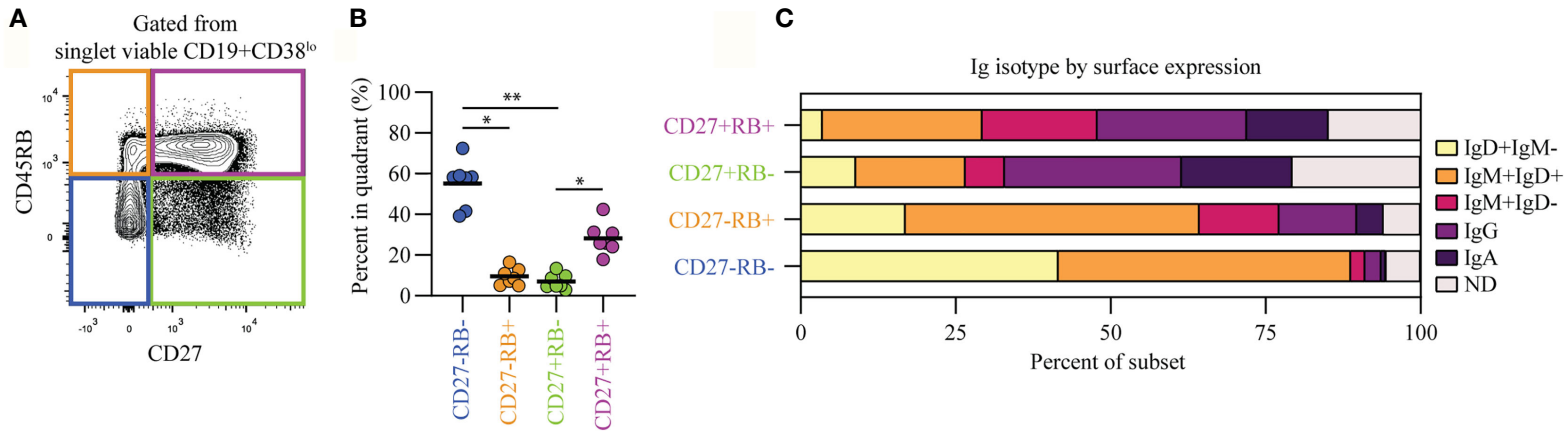
B cells within subsets separated based on CD45RB glycosylation and CD27 expression feature differential Ig isotype usage. **(A)** Representative biaxial CD45RB/CD27 plot gated from singlet viable CD19+CD38^lo^ cells (**Figure S1A**). **(B)** Percent of B cells in each quadrant, as in **(A)**, for seven biological replicates (healthy adults) each composed of two technical replicates over three independent experiments. **(C)** I_g_-isotype usage per quadrant. ND, not determined. Black lines depict mean values. Statistical differences were determined using a Friedman analysis of variance and Dunn’s multiple comparison test. *p < 0.05, **p < 0.01.

Human peripheral blood CD19+CD38^lo^ B cells (**Figure S1A**, pre-gating strategy) can be segregated into four populations based on their CD45RB glycosylation (RB) and CD27 expression levels (**Figure 1A**). The CD27-RB- (55%) and CD27+RB+ (28%) populations are more prevalent compared to CD27-RB+ (9.5%) and CD27+RB- (7%) (**Figure 1B**). Within these populations differential Ig isotype usage was observed, with CD27+ cells enriched for IgG and IgA and CD27- cells mostly being IgM+ and/or IgD+ (**Figure 1C**). Within CD27+ populations, the Ig isotype usage was quite similar, despite cells being RB+ or RB-. By contrast, within CD27- populations, there was an increased prevalence of all IgD- subsets within the RB+ population. By isotype-specific gating around 13% of the CD19+ cells were left unannotated, in line with a recent study (14). These cells are not mislabeled IgE+ B cells as these are extremely rare in healthy peripheral blood (20). The Ig^lo^ cells were found across all four populations and likely represent a mixture of isotypes found on cells that recently underwent class-switching. Thus, the CD27-RB+ population features more isotype-switched cells than the CD27-RB- population, a difference not observed within CD27+ populations.

### CD45RB Glycosylation Marks Antigen-Primed B Cells Independent of CD27 Expression

**Figure 2:**
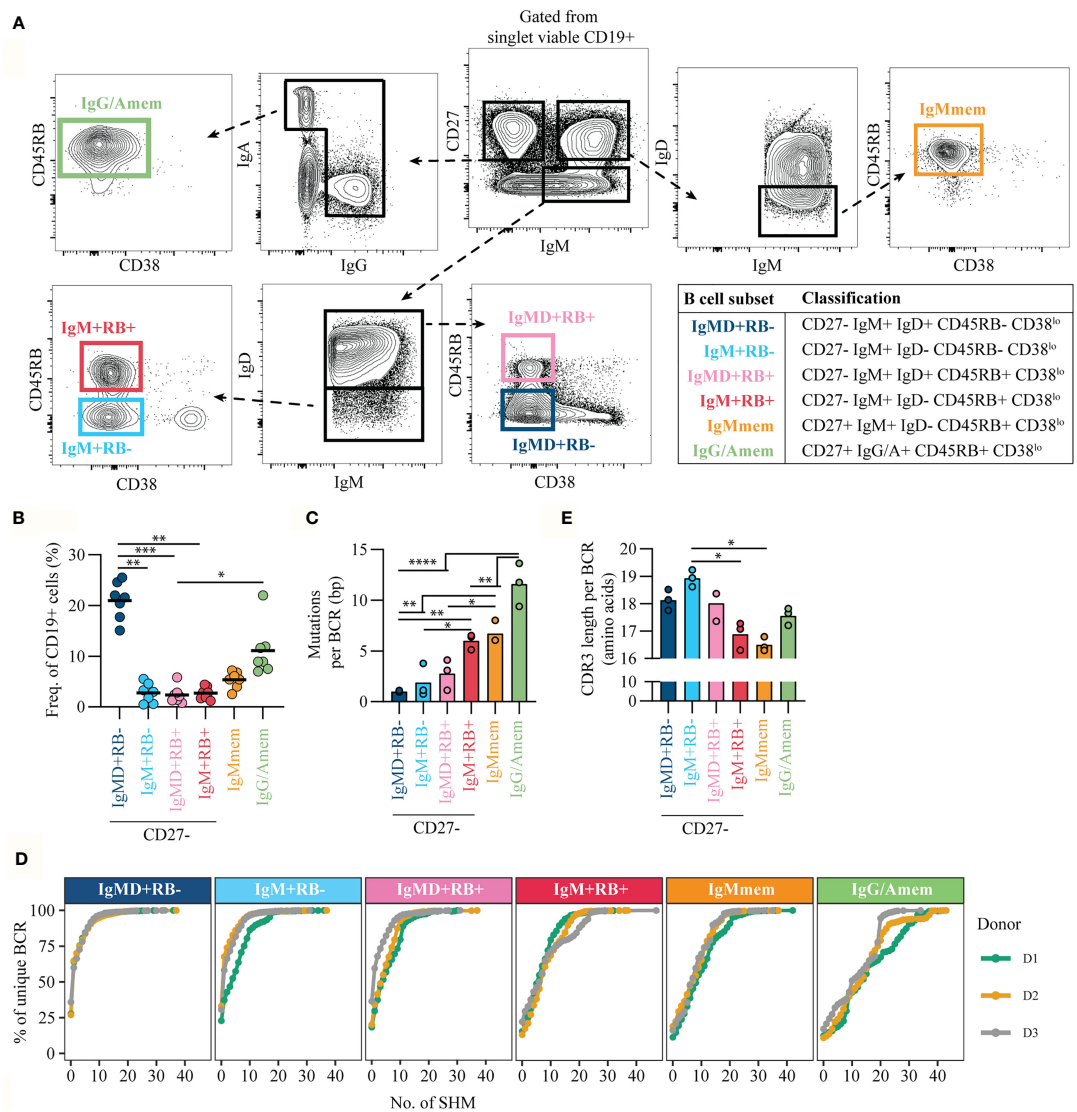
CD45RB glycosylation marks antigen-primed B cells independent of CD27 expression. **(A)** Representative gating strategy for cell-sorting B cell subsets from healthy donor peripheral blood CD19+ pre-isolated B cells by flow cytometry. Gated from singlet viable CD19+ cells (**Figure S1B**). Table shows the classification of subsets. **(B)** Frequency of subsets within CD19+ cells, for seven biological replicates each composed of two technical replicates over three independent experiments. **(C)** Mean number of mutations per Ig gene (healthy adults, n = 3) and **(D)** Cumulative frequency distribution of mutations in the Ig repertoire in the different B cell subsets. Different colored lines represent different donors. **(E)** mean V_H_-CDR3 length from cell-sorted B cell subsets (healthy adults, n = 3). Bars and black lines depict mean values. Statistical differences were determined using a Friedman analysis of variance and Dunn’s multiple comparison test. *p < 0.05, **p < 0.01, ***p < 0.001, ****p < 0.0001.

To refine discrimination of CD27/RB populations we included Ig isotype expression for enhanced characterization. In the current study we focused on characterizing the non-isotype switched (IgM and IgD) B cells but excluded CD27-IgD+IgM- B cells that represent a population of anergic B cells that recognize self-antigens (21–23). Based on the expression of CD27, CD45RB glycosylation and IgM and IgD we discriminated four CD27- populations (**Figure 2A**, **Figure S1B**): CD27-RB-IgM+IgD+ [IgMD+RB-], CD27-RB-IgM+IgD- [IgM+RB-], CD27-RB+IgM+IgD+ [IgMD+RB+], and CD27-RB+IgM+IgD- [IgM+RB+]. In addition, two CD27+RB+ populations were included: IgM+IgD- memory B cells [IgMmem] and isotype-switched memory B cells [IgG/Amem]. Subset classifications are depicted (table, **Figure 2A**). All subsets were gated on being CD38^lo^ to exclude CD38+ transitional B cells and CD38+ ASCs. Combined the CD27- subsets make up ~30% of the adult human peripheral blood B cell population with the largest fraction being IgMD+RB- cells (n = 7, **Figure 2B**). To investigate the levels of somatic hypermutation in the six different subsets we performed next-generation sequencing of IGHV genes (n = 3). We observed increased somatic mutations in V_H_-regions of both memory B cell subsets (IgMmem and IgG/Amem), in line with previous studies (24, 25). V_H_-mutations were virtually absent for IgMD+RB- cells and not significantly increased for both IgM+RB- and IgMD+RB+ subsets compared to IgMD+RB- cells (**Figure 2C**). Remarkably, IgM+RB+ cells had comparable mutation counts as IgMmem, but less than IgG/Amem. Similar results were observed when analyzing the entire cumulative distribution of mutations in the Ig repertoire (**Figure 2D**). In addition, IgM+RB+ cells displayed a reduction in V_H_-CDR3 length (25, 26), also similar to IgMmem (**Figure 2E**). Taken together, IgM+RB+ cells show characteristic that associate with being antigen-primed despite being CD27-.

### CD45RB Glycosylation, Combined With Ig Isotype, Identifies Functionally Distinct B Cell Subsets

Apart from expression of mutated V_H_ genes, memory B cells can be distinguished from naive B cells by more efficient proliferation and differentiation into antibody-secreting cells (ASCs) upon stimulation (26, 27). Given that the IgM+RB+ subset carried somatic mutations they may also functionally resemble memory B cells. Therefore, we studied their *in vitro* functionality, and of the other CD27- subsets, using T cell-dependent (TD) and -independent (TI) stimulation and analyzed survival, differentiation and isotype-switching potential.

**Figure 3:**
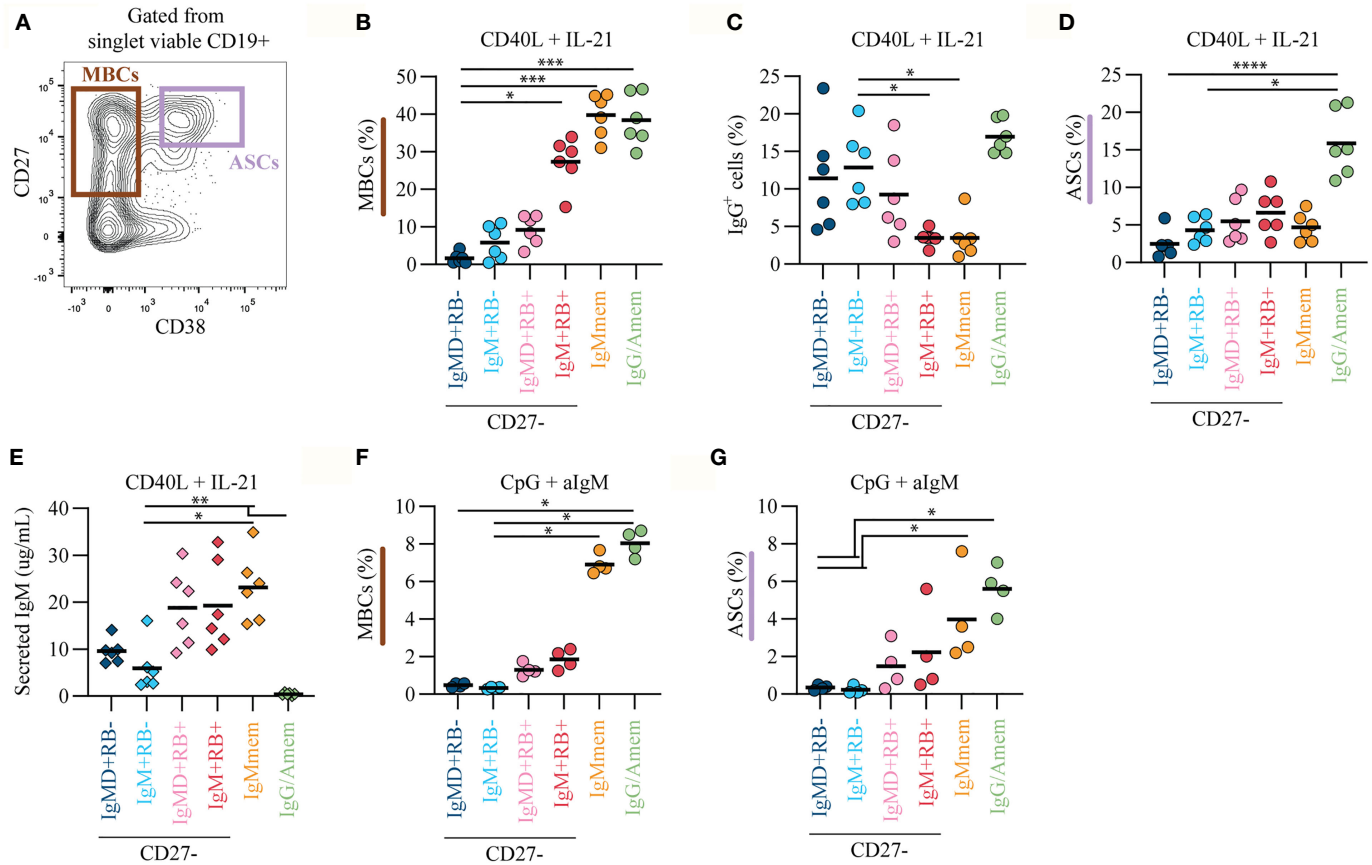
CD45RB glycosylation, combined with Ig isotype, marks functionally distinct B cell subsets. B cell subsets were cultured with TD-stimulation of CD40L-expressing 3T3s in the presence of IL-21 (50ng/ml) for 10 days (six biological replicates each consisting of two technical replicates over two independent experiments). **(A)** Representative biaxial CD27/CD38 FACS plot after 10 days of TD culture. The formation of **(B)** MBCs (CD27+CD38-) **(C)** IgG+ B cells, and **(D)** ASCs (CD27+CD38+) was measured using flow cytometry. **(E)** Cumulative IgM secretion measured in culture supernatants using ELISA at day 10 (n = 6). B cell subsets were cultured with TI-stimulation of CpG ODN (0.1μM) and anti-IgM-F(ab’)_2_ (1μg/ml) for 7 days (four biological replicates each consisting of two technical replicates). The formation of **(F)** MBCs and **(G)** ASCs was measured using flow cytometry. Black lines depict mean values. Statistical differences were determined using a Friedman analysis of variance and Dunn’s multiple comparison test. *p < 0.05, **p < 0.01, ***p < 0.001, ****p < 0.0001.

The six B cell subsets were cell-sorted and cultured on CD40L-expressing cells in the presence of IL-21 for 10 days. Survival and/or proliferation was similar for most subsets (**Figure S2A**). Compared to IgMD+RB- cells, IgM+RB+ cells showed increased differentiation towards activated and/or memory-like B cells (MBCs, CD27+CD38-, **Figures 3A, B**). Within IgMmem and IgG/Amem populations, that were sorted as being CD27+, we observed downregulation of CD27 expression, a phenomenon previously observed by others upon *in vitro* culture of memory B cells (28, 29). Interestingly, IgM+RB+ cells, but not IgMD+RB+ cells, had poor IgG switching potential, a feature shared with IgMmem (**Figure 3C**). The addition of IL-4, a TD cytokine known to enhance isotype switch to IgG, had limited additional effects and did not alleviate the reduced IgG switch observed for IgM+RB+ cells (**Figures S2B–F**). Furthermore, the IgM+RB+ subset showed increased differentiation into functional ASCs (CD27+CD38+, **Figures 3A, D, E** and **Figure S2G**), however, this was less in comparison to IgG/Amem. IgMD+RB+ cells, although not significant, did show a trend in increased MBC and ASC formation compared to IgMD+RB- and IgM+RB- cells, but less in comparison to IgM+RB+ cells (**Figures 3B, D, E**). Differentiation of B cells also occurs during T cell-independent (TI) immune responses that generally results in the formation of ASCs that secrete low-affinity IgM antibodies (30). Since IgM+RB+ cells showed reduced isotype switch to IgG upon *in vitro* TD stimulation we studied their functionality, and of the other CD27- subsets, upon *in vitro* TI stimulation. After 7 days of TI stimulation with CpG ODN (TLR9 activator) and anti-IgM, IgM+RB+ and IgMD+RB+ subsets, but not IgM+RB- and IgMD+RB- subsets, were able to differentiate into memory-like B cells (**Figure 3F**), albeit, much less when compared to TD stimulation. Furthermore, IgM+RB+ and IgMD+RB+ subsets showed a trend towards increased differentiation into functional ASCs as was observed for IgMmem and IgG/Amem, albeit to various degrees (**Figures 3G**, **S3A**), and with limited isotype switching (**Figure S3B**). Subsets responded in a similar fashion when stimulated with R848 (TLR7/TLR8 agonist) and anti-IgM for 7 days (**Figure S4**). Thus, different from IgMD+RB+, IgM+RB- and IgMD+RB- subsets, IgM+RB+ cells showed a higher propensity for MBC differentiation with an IgM isotype commitment.

## Discussion

Here, by using a novel combination of markers including CD45RB glycosylation, CD27 and IgM/IgD isotype expression we segregated human peripheral blood B cell subsets and investigated their IGHV repertoire and *in vitro* functionality. **Table 1** summarizes for each of the studied B cell subset their V_H_ mutation status and *in vitro* functionality.

**Table 1:** Summary of B cell subset discriminating factors.

B cell subset	V_H_ mutations	MBC TD-stim.	IgG+ TD-stim.	IgM secr. TD-stim.	MBC TI-stim.
**IgMD+RB-**	low	**-**	**+**	**+/-**	**-**
**IgM+RB-**	low	**-**	**+**	**+/-**	**-**
**IgMD+RB+**	low	**-**	**+**	**+**	**-**
**IgM+RB+**	int	**+**	**-**	**+**	**-**
**IgMmem**	int	**+**	**-**	**+**	**+**
**IgG/Amem**	high	**+**	**+**	**-**	**+**

MBC, memory B cell differentiation; TD, T cell-dependent; TI, T cell-independent.

+, good response; -, poor response..

IgMD+ RB- cells displayed virtually absent levels of somatic hypermutation, indicating that this population corresponds to “classical” naive B cells. IgM+RB- and IgMD+RB+ cells were both in repertoire and *in vitro* functionality similar to IgMD+RB- cells. Because IgM+RB- cells were gated on being CD38^lo^ we do not assume that they represent human T1 B cells that are often characterized by a CD24^hi^CD38^hi^ phenotype (31). Circulating IgM+RB+ cells carried mutated V_H_ genes and reduced CDR3 length which implies previous antigen-priming. This suggests that these cells are at a later stage of development with regards to affinity maturation as compared to the other CD27- subsets. In line, IgM+RB+ cells also showed improved ability to develop into functional ASCs using TI and TD *in vitro* stimulations. IgMD+RB+ cells, although not significant and less in comparison to IgM+RB+, did show a trend in increased MBC and ASC formation compared to IgMD+RB- and IgM+RB- cells. These data indicate that CD27-RB+ populations fall within the continuum of naive B cells that progress towards memory B cells and ASCs and do not represent a population of exhausted memory B cells with downregulated CD27, as was proposed previously (5). In addition, the absence of CD45RB glycosylation to define ‘true’ human naive B cells may advance current strategies using CD27 and IgD. Even though using the glycosylation epitope on CD45RB helps to identify antigen-primed B cells among CD27- cell, there is, however, a population of CD27+ cells, presumed memory B cells, that are RB-, meaning that glycosylation of CD45RB does not mark all antigen primed B cells.

We show that IgM+RB+ cells have properties in common with CD27+IgM+IgD-RB+ cells (IgMmem). IgM+RB+ cells seem to have an early lineage commitment to the IgM isotype as they showed reduced ability to isotype-switch to IgG, similar to IgMmem but in steep contrast to the other CD27- subsets. Moreover, CD27 expression was higher on IgM+RB+ cells after 10 days of TD culture compared to the other CD27- subsets which shows that with the same level of stimulation these cells had enhanced capacity to differentiate towards a memory phenotype. IgM memory B cells are important for the generation of a broadly cross-reactive memory repertoire with lower affinity BCRs to protect against a plethora of pathogens and their variants (32–34). The early lineage commitment of IgM+RB+ cells to the IgM isotype might represent a mechanism to ensure replenishment of the IgM memory pool for lasting B cell immunity. In contrast, the efficient IgG isotype-switching seen for IgMD+RB+ cells suggests this subset has more plasticity in B cell fate decision. Thus, within RB+ cells, differential expression of IgM and IgD, discriminates two functionally distinct B cell subsets. In line, for CD27+RB+ subsets, only IgG/Amem, but not IgMmem, outperformed both CD27-RB+ subsets in ASC differentiation. This suggest that expression of just CD27 or CD45RB glycosylation upon B cell activation is not indicative for enhanced ASC differentiation, but also relies on the Ig isotype. The notion that the Ig isotype imprints the subsequent fate of the B cell upon recall responses is consistent with data obtained from mouse and human *in vitro* models indicating that in general the IgM memory B cells re-entered germinal centers to undergo multiple rounds of expansion, somatic hypermutation and selection, whereas IgG memory B cells preferentially differentiated into ASCs (35–37).

In this study we did not analyze memory subsets with differential expression of IgM and IgD and only included the CD27+IgM+IgD-RB+ subset (IgMmem). For CD27+IgM+IgD+ cells it has been described that they have fewer V_H_-gene mutation than CD27+IgM+IgD- and class-switched CD27+ cells but more mutations than naive (CD27-IgM+IgD+) and CD27+IgM-IgD+ cells (38). CD27+IgM-IgD+ cells only make up a small fraction of CD27+ cells (7.5%, **Figure 1C**). Within CD27+RB+ cells, CD27+IgM+IgD+ cells make up around 25% cells and are present to a lesser degree within the CD27+RB- population (16%, **Figure 1C**). Whether, the presence or absence of glycosylated CD45RB on CD27+ cells with differential IgM and IgD expression impacts the level of detectible V_H_-gene mutations and *in vitro* functionality remains unclear.

Previous studies reported increased frequencies of CD27-RB+ cells that expressed high levels of IgM in young children and early after hematopoietic stem cell transplantation (11). Unfortunately, the dual IgM/IgD expression on these cells was not determined. Therefore, we cannot assign with certainty whether these are CD27-IgM+RB+ or the CD27-IgMD+RB+ cells. As these cells have increased frequency in children it may be more likely that these are the CD27-IgMD+RB+ cells described here that have fewer mutations and showed a naive-like behavior. However, cord blood and fetal tissues also accommodate a small population of mutated CD27+IgM+IgD+ B cells (39, 40). These subsets may represent a first-line defense mechanism that respond rapidly upon both TD and TI activation.

The findings presented here provide novel insights in the phenotypic and functional progression from naive B cells *via* CD27-RB+ cells to memory B cells in human peripheral blood. In conclusion, glycosylation of CD45RB is indicative for antigen-primed B cells, which are, dependent on the Ig isotype, functionally distinct.

## Data Availability Statement

The datasets presented in this study can be found in online repositories. The names of the repository/repositories and accession number(s) can be found below: NCBI, accession code: PRJNA816414.

## Ethics Statement

Ethical review and approval was not required for the study on human participants in accordance with the local legislation and institutional requirements. The patients/participants provided their written informed consent to participate in this study.

## Author Contributions

JK, SP, ST, PvS, MB, and TR designed research. JK, ST, IPK, and ND performed research. JK, SP, and ND analyzed data. JK, SP, and TR wrote the paper. All authors critically reviewed the manuscript, gave final approval of the version to be published, and agreed to be accountable for all aspects of the work ensuring that questions related to the accuracy or integrity of any part of the work are appropriately investigated and resolved.

## Funding

This study was supported by Landsteiner Foundation for Blood Transfusion Research (Grant1626).

## Conflict of Interest

The authors declare that the research was conducted in the absence of any commercial or financial relationships that could be construed as a potential conflict of interest.

## Publisher’s Note

All claims expressed in this article are solely those of the authors and do not necessarily represent those of their affiliated organizations, or those of the publisher, the editors and the reviewers. Any product that may be evaluated in this article, or claim that may be made by its manufacturer, is not guaranteed or endorsed by the publisher.

## References

[1] Sanz I Wei C Lee FEH Anolik J. Phenotypic and Functional Heterogeneity of Human Memory B Cells. Semin Immunol (2008) 20(1):67–82. doi: 10.1016/j.smim.2007.12.006 18258454 PMC2440717

[2] Berkowska MA Driessen GJA Bikos V Grosserichter-Wagener C Stamatopoulos K Cerutti A. Human Memory B Cells Originate From Three Distinct Germinal Center-Dependent and -Independent Maturation Pathways. Blood (2011) 118(8):2150–8. doi: 10.1182/blood-2011-04-345579 PMC3342861 21690558

[3] Dunn-Walters DK Isaacson PG Spencer J. MGZ of Human Spleen Is a Reservoir of Memory. J Exp Med (1995) 182(August):559–66. doi: 10.1084/jem.182.2.559 PMC2192131 7629512

[4] van Gent R van Tilburg CM Nibbelke EE Otto SA Gaiser JF Janssens-Korpela PL. Refined Characterization and Reference Values of the Pediatric T- and B-Cell Compartments. Clin Immunol (2009) 133(1):95–107. doi: 10.1016/j.clim.2009.05.020 19586803

[5] Colonna-Romano G Bulati M Aquino A Pellicanò M Vitello S Lio D. A Double-Negative (IgD-CD27-) B Cell Population Is Increased in the Peripheral Blood of Elderly People. Mech Ageing Dev (2009) 130(10):681–90. doi: 10.1016/j.mad.2009.08.003 19698733

[6] Wei C Anolik J Cappione A Zheng B Pugh-Bernard A Brooks J. A New Population of Cells Lacking Expression of CD27 Represents a Notable Component of the B Cell Memory Compartment in Systemic Lupus Erythematosus. J Immunol (2007) 178(10):6624–33. doi: 10.4049/jimmunol.178.10.6624 17475894

[7] Moir S Ho J Malaspina A Wang W DiPoto AC O’Shea MA. Evidence for HIV-Associated B Cell Exhaustion in a Dysfunctional Memory B Cell Compartment in HIV-Infected Viremic Individuals. J Exp Med (2008) 205(8):1797–805. doi: 10.1084/jem.20072683 PMC2525604 18625747

[8] Rojas OL Narváez CF Greenberg HB Angel J Franco MA. Characterization of Rotavirus Specific B Cells and Their Relation With Serological Memory. Virology (2008) 380(2):234–42. doi: 10.1016/j.virol.2008.08.004 PMC2582161 18789807

[9] Zuccarino-Catania GV Sadanand S Weisel FJ Tomayko MM Meng H Kleinstein SH. CD80 and PD-L2 Define Functionally Distinct Memory B Cell Subsets That Are Independent of Antibody Isotype. Nat Immunol (2014) 15(7):631–7. doi: 10.1038/ni.2914 PMC4105703 24880458

[10] Koethe S Zander L Koster S Annan A Ebenfelt A Spencer J. Pivotal Advance: CD45RB Glycosylation Is Specifically Regulated During Human Peripheral B Cell Differentiation. J Leukoc Biol (2011) 90(1):5–19. doi: 10.1189/jlb.0710404 21278234

[11] Bemark M Friskopp L Saghafian-Hedengren S Koethe S Fasth A Abrahamsson J. A Glycosylation-Dependent CD45RB Epitope Defines Previously Unacknowledged CD27-IgMhigh B Cell Subpopulations Enriched in Young Children and After Hematopoietic Stem Cell Transplantation. Clin Immunol (2013) 149(3 PB):421–31. doi: 10.1016/j.clim.2013.08.011 24211716

[12] Jackson SM Harp N Patel D Wulf J Spaeth ED Dike UK. Key Developmental Transitions in Human Germinal Center B Cells Are Revealed by Differential CD45RB Expression. Blood (2009) 113(17):3999–4007. doi: 10.1182/blood-2008-03-145979 19059880 PMC2673126

[13] Zhao Y Uduman M Siu JHY Tull TJ Sanderson JD Wu YCB. Spatiotemporal Segregation of Human Marginal Zone and Memory B Cell Populations in Lymphoid Tissue. Nat Commun (2018) 9(1):3857. doi: 10.1038/s41467-018-06089-1 30242242 PMC6155012

[14] Glass DR Tsai AG Oliveria JP Hartmann FJ Kimmey SC Calderon AA. An Integrated Multi-Omic Single-Cell Atlas of Human B Cell Identity. Immunity (2020) 53(1):217–232.e5. doi: 10.1016/j.immuni.2020.06.013 32668225 PMC7369630

[15] Vander Heiden JA Yaari G Uduman M Stern JNH O’connor KC Hafler DA. PRESTO: A Toolkit for Processing High-Throughput Sequencing Raw Reads of Lymphocyte Receptor Repertoires. Bioinformatics (2014) 30(13):1930–2. doi: 10.1093/bioinformatics/btu138 PMC4071206 24618469

[16] Lefranc M. IMGT, the International ImMunoGeneTics Information System. Cold Spring Harb Protoc (2011) 6):595–603. doi: 10.1101/pdb.top115 21632786

[17] Brochet X Lefranc M Giuidicelli V. IMGT / V-QUEST: The Highly Customized and Integrated System for IG and TR Standardized V-J and V-D-J Sequence Analysis. Nucleic Acids Res (2008) 36:503–8. doi: 10.1093/nar/gkn316 PMC2447746 18503082

[18] Gupta NT Vander Heiden JA Uduman M Gadala-Maria D Yaari G Kleinstein SH. Change-O: A Toolkit for Analyzing Large-Scale B Cell Immunoglobulin Repertoire Sequencing Data. Bioinformatics (2015) 31(20):3356–8. doi: 10.1093/bioinformatics/btv359 PMC4793929 26069265

[19] Unger PPA Verstegen NJM Marsman C Jorritsma T Rispens T Ten Brinke A. Minimalistic *In Vitro* Culture to Drive Human Naive B Cell Differentiation Into Antibody-Secreting Cells. Cells (2021) 10(5):1183. doi: 10.3390/cells10051183 34066151 PMC8151070

[20] Jiménez-Saiz R Ellenbogen Y Bruton K Spill P Sommer DD Lima H. Human BCR Analysis of Single-Sorted, Putative IgE+ Memory B Cells in Food Allergy. J Allergy Clin Immunol (2019) 144(1):336–339.e6. doi: 10.1016/j.jaci.2019.04.001 30959060 PMC7010227

[21] Gutzeit C Chen K Cerutti A. The Enigmatic Function of IgD: Some Answers at Last. Eur J Immunol (2018) 48(7):1101–13. doi: 10.1002/eji.201646547 PMC6033660 29733429

[22] Quách TD Manjarrez-Orduño N Adlowitz DG Silver L Yang H Wei C. Anergic Responses Characterize a Large Fraction of Human Autoreactive Naive B Cells Expressing Low Levels of Surface IgM. J Immunol (2011) 186(8):4640–8. doi: 10.4049/jimmunol.1001946 PMC3095097 21398610

[23] Merrell KT Benschop RJ Gauld SB Aviszus K Decote-Ricardo D Wysocki LJ. Identification of Anergic B Cells Within a Wild-Type Repertoire. Immunity (2006) 25(6):953–62. doi: 10.1016/j.immuni.2006.10.017 17174121

[24] Wu YC Kipling D Leong HS Martin V Ademokun AA Dunn-Walters DK. High-Throughput Immunoglobulin Repertoire Analysis Distinguishes Between Human IgM Memory and Switched Memory B-Cell Populations. Blood (2010) 116(7):1070–8. doi: 10.1182/blood-2010-03-275859 PMC2938129 20457872

[25] DeKosky BJ Lungu OI Park D Johnson EL Charab W Chrysostomou C. Large-Scale Sequence and Structural Comparisons of Human Naive and Antigen-Experienced Antibody Repertoires. Proc Natl Acad Sci USA (2016) 113(19):E2636–45. doi: 10.1073/pnas.1525510113 PMC4868480 27114511

[26] Wu YCB Kipling D Dunn-Walters DK. The Relationship Between CD27 Negative and Positive B Cell Populations in Human Peripheral Blood. Front Immunol (2011) 2(DEC):1–12. doi: 10.3389/fimmu.2011.00081 22566870 PMC3341955

[27] Deenick EK Avery DT Chan A Berglund LJ Ives ML Moens L. Naive and Memory Human B Cells Have Distinct Requirements for STAT3 Activation to Differentiate Into Antibody-Secreting Plasma Cells. J Exp Med (2013) 210(12):2739–53. doi: 10.1084/jem.20130323 PMC3832925 24218138

[28] Racanelli V Frassanito MA Leone P Galiano M De Re V Silvestris F. Antibody Production and *In Vitro* Behavior of CD27-Defined B-Cell Subsets: Persistent Hepatitis C Virus Infection Changes the Rules. J Virol (2006) 80(8):3923–34. doi: 10.1128/JVI.80.8.3923-3934.2006 PMC1440441 16571809

[29] van Asten SD Unger P-P Marsman C Bliss S Jorritsma T Thielens NM. Soluble FAS Ligand Enhances Suboptimal CD40L/IL-21–Mediated Human Memory B Cell Differentiation Into Antibody-Secreting Cells. J Immunol (2021) 207(2):449–58. doi: 10.4049/jimmunol.2001390 34215657

[30] Obukhanych TV Nussenzweig MC. T-Independent Type II Immune Responses Generate Memory B Cells. J Exp Med (2006) 203(2):305–10. doi: 10.1084/jem.20052036 PMC2118207 16476769

[31] Sims GP Ettinger R Shirota Y Yarboro CH Illei GG Lipsky PE. Identification and Characterization of Circulating Human Transitional B Cells. Blood (2005) 105(11):4390–8. doi: 10.1182/blood-2004-11-4284 PMC1895038 15701725

[32] Burton BR Tennant RK Love J Titball RW Wraith DC White HN. Variant Proteins Stimulate More IgM+ GC B-Cells Revealing a Mechanism of Cross-Reactive Recognition by Antibody Memory. Elife (2018) 7:1–14. doi: 10.7554/eLife.26832 PMC5959717 29709214

[33] Keating R Hertz T Wehenkel M Harris TL Benjamin A Mcclaren JL. Protective Immunity To Lethal Influenza Infections. Nat Immunol (2013) 14(12):1266–76. doi: 10.1038/ni.2741 PMC3883080 24141387

[34] Weill JC Reynaud CA. IgM Memory B Cells: Specific Effectors of Innate-Like and Adaptive Responses. Curr Opin Immunol (2020) 63:1–6. doi: 10.1016/j.coi.2019.09.003 31639539 PMC6942539

[35] Dogan I Bertocci B Vilmont V Delbos F Mégret J Storck S. Multiple Layers of B Cell Memory With Different Effector Functions. Nat Immunol (2009) 10(12):1292–9. doi: 10.1038/ni.1814 19855380

[36] Pape KA Taylor JJ Maul RW Gearhart PJ Jenkins MK. Different B Cell Populations Mediate Early and Late Memory During an Endogenous Immune Response. Science (2011) 331(6021):1203–7. doi: 10.1126/science.1201730 PMC3993090 21310965

[37] Seifert M Przekopowitz M Taudien S Lollies A Ronge V Drees B. Functional Capacities of Human Igm Memory B Cells in Early Inflammatory Responses and Secondary Germinal Center Reactions. Proc Natl Acad Sci USA. (2015) 112(6):E546–55. doi: 10.1073/pnas.1416276112 PMC4330750 25624468

[38] Bautista D Vásquez C Ayala-Ramírez P Téllez-Sosa J Godoy-Lozano E Martínez-Barnetche J. Differential Expression of IgM and IgD Discriminates Two Subpopulations of Human Circulating IgM+IgD+CD27+ B Cells That Differ Phenotypically, Functionally, and Genetically. Front Immunol (2020) 11(May):1–19. doi: 10.3389/fimmu.2020.00736 32435242 PMC7219516

[39] Weller S Faili A Garcia C Braun MC Le Deist F De Saint Basile G. CD40-CD40L Independent Ig Gene Hypermutation Suggests a Second B Cell Diversification Pathway in Humans. Proc Natl Acad Sci USA (2001) 98(3):1166–70. doi: 10.1073/pnas.98.3.1166 PMC14726 11158612

[40] Scheeren FA Nagasawa M Weijer K Cupedo T Kirberg J Legrand N. T Cell-Independent Development and Induction of Somatic Hypermutation in Human IgM+IgD+CD27+ B Cells. J Exp Med (2008) 205(9):2033–42. doi: 10.1084/jem.20070447 PMC2526198 18695003

