# Supplementary material for: CD45RB Glycosylation and Ig Isotype Define Maturation of Functionally Distinct B Cell Subsets in Human Peripheral Blood
Source: Front Immunol. 2022 Apr 28;13:891316. doi: 10.3389/fimmu.2022.891316 (PMC9095956; doi:10.3389/fimmu.2022.891316)
Supplement: Supplementary file 1 [file DataSheet_1.pdf]

Supplementary Figures

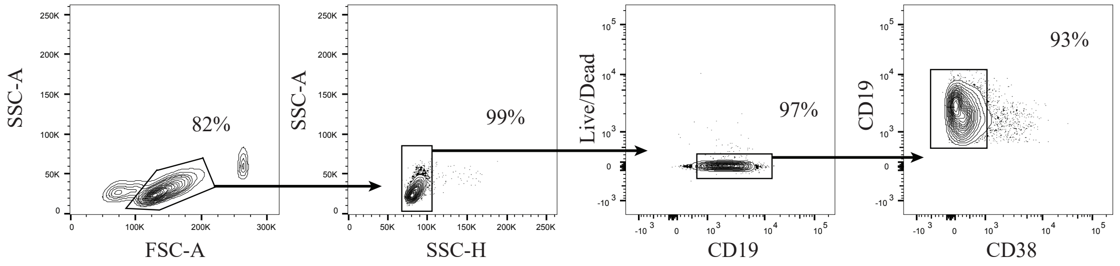

Figure S1A. Cryopreserved CD19+ magnetic bead isolated B cells were pre-gated to obtain singlet viable CD19+CD38^{lo} cells for flow cytometry (fig. 1A).

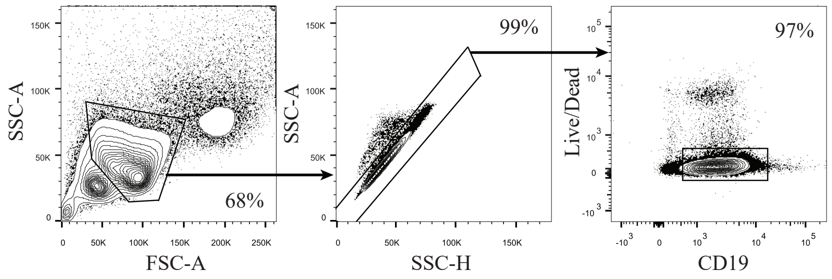

Figure S1B. Cryopreserved CD19+ magnetic bead isolated B cells were pre-gated to obtain singlet viable CD19+ cells for cell-sorting (fig. 2A).

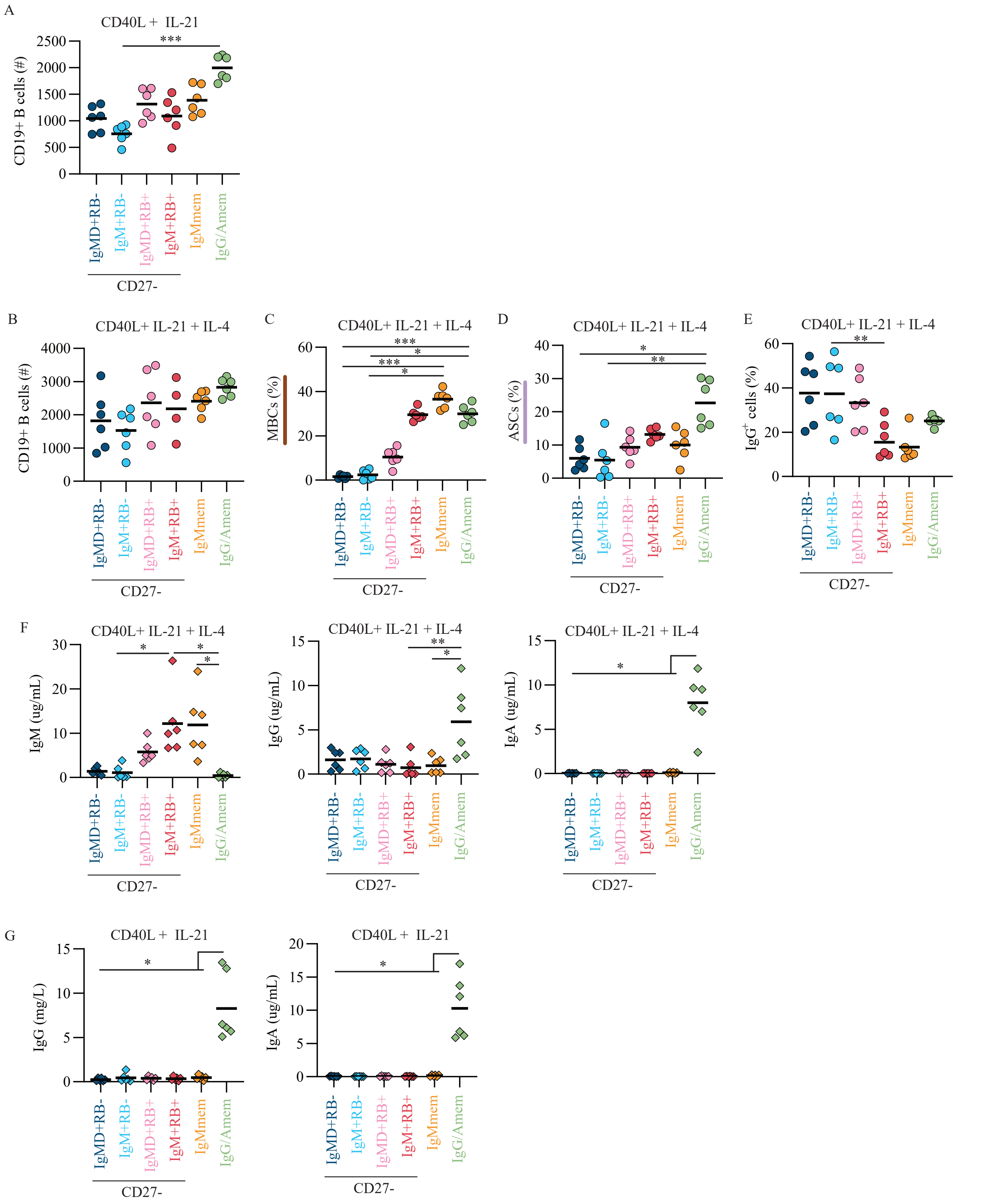

Figure S2. B cell subsets were cultured with TD stimulation for 10 days. 3T3-CD40L expressing cells and IL-21 (50ng/ml) for 10 days. (**A**) Survival measured in subsets cultured with 3T3-CD40L expressing cells and IL-21 (50ng/ml) by flow cytometry (six biological replicates each consisting of two technical replicates over 2 independent experiments). B cell subsets cultured with CD40L-expressing 3T3s for 10 days in the presence of IL-21 (50ng/ml) and IL-4 (25ng/ml). (**B**) Survival, (**C**) frequencies of MBCs, (**D**) ASCs, and (**E**) IgG+ cells were measured using flow cytometry (six biological replicates each consisting of two technical replicates over 2 independent experiments). Ig secretion in TD-cultures with IL-21 and IL-4 (**F**) and IL-21 (**G**) was measured in culture supernatants by ELISA. Black lines depict mean values. Statistical differences were determined using a Friedman analysis of variance and Dunn's multiple comparison test. * $p < 0.05$, ** $p < 0.01$, *** $p < 0.001$, **** $p < 0.0001$.

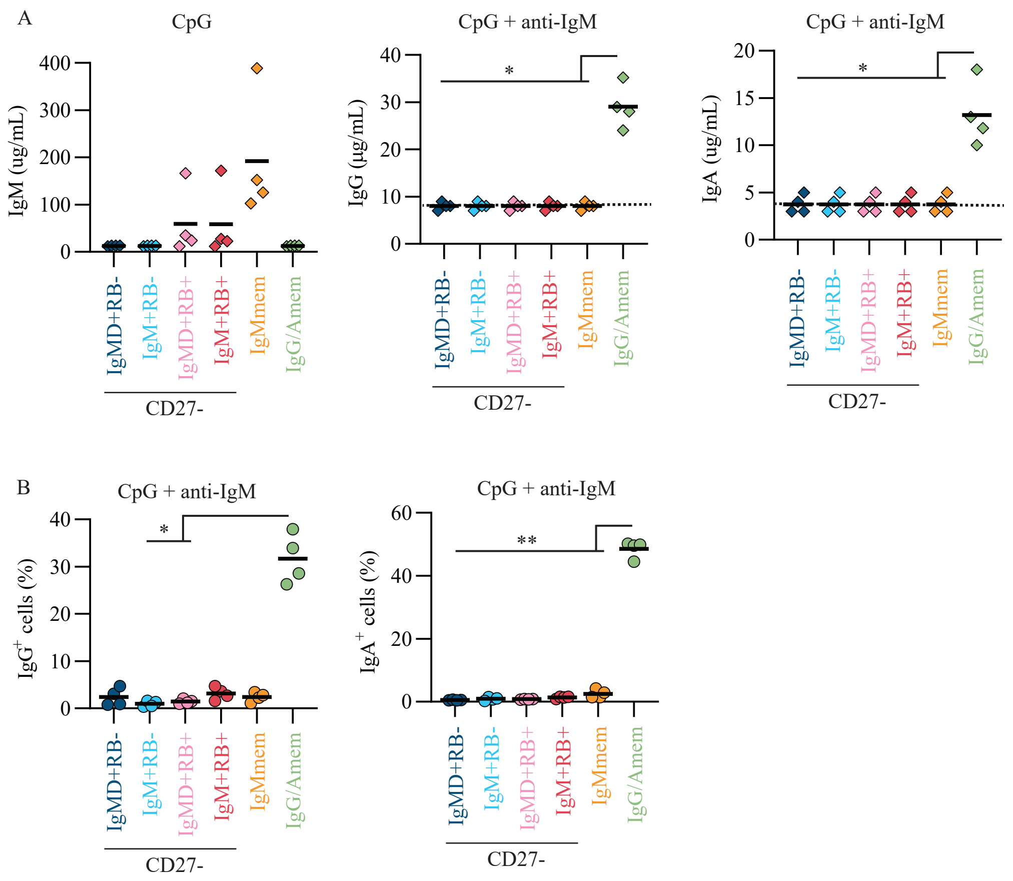

Figure S3. B cell subsets were cultured with CpG (0.1μM) and anti-IgM-F(ab')$_2$ (1μg/ml) for 7 days. (**A**) Ig secretion was measured in culture supernatants by ELISA (four biological replicates each consisting of two technical replicates). (**B**) IgG and IgA isotype switching was measured by flow cytometry. Black lines depict mean values. Statistical differences were determined using a Friedman analysis of variance and Dunn's multiple comparison test. * $p < 0.05$, ** $p < 0.01$, *** $p < 0.001$, **** $p < 0.0001$.

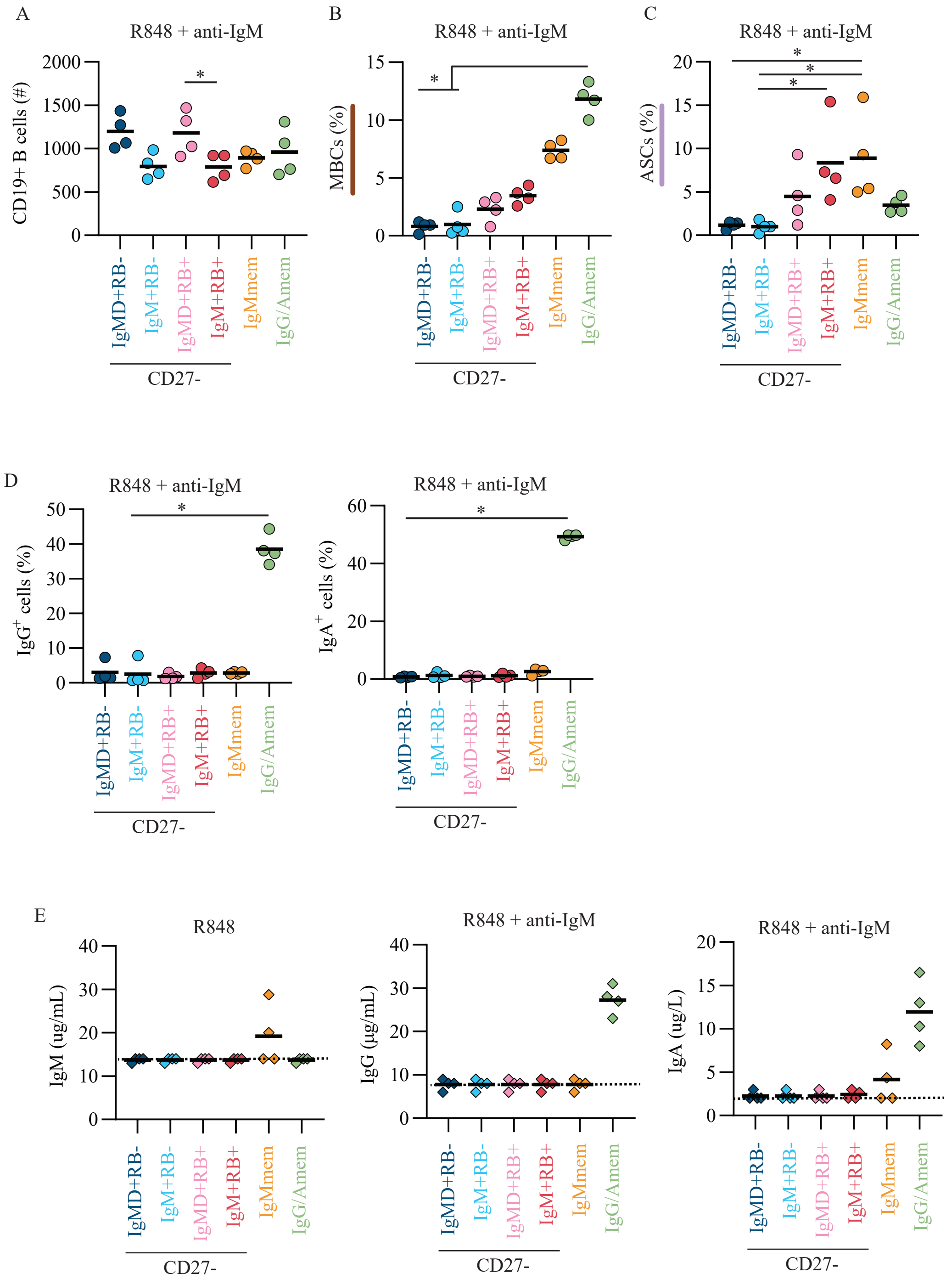

Figure S4. B cell subsets cultured with R848 (1µM) and anti-IgM-F(ab')$_2$ (1µg/ml) for 7 days. (**A**) Survival, (**B**) frequencies of MBCs, (**C**) ASCs (**D**) and IgG+ and IgA+ cells were measured using flow cytometry in four biological replicates each consisting of two technical replicates. (**E**) Ig secretion was measured after 7 days by ELISA. Dashed lines represent ELISA detection limits. Black lines depict mean values. Statistical differences were determined using a Friedman analysis of variance and Dunn's multiple comparison test. * $p < 0.05$, ** $p < 0.01$, *** $p < 0.001$, **** $p < 0.0001$.
